# Children with Kaposi Sarcoma in Two Southern African Hospitals: Clinical Presentation, Management, and Outcome

**DOI:** 10.1155/2013/213490

**Published:** 2013-12-11

**Authors:** G. P. De Bruin, D. C. Stefan

**Affiliations:** Department of Pediatrics and Child Health, Tygerberg Children's Hospital, Stellenbosch University, P.O. Box 19063, Tygerberg, Cape Town 7550, South Africa

## Abstract

*Introduction*. In 2010 more than 3 million children with human immunodeficiency virus (HIV) were living in Sub-Saharan Africa. The AIDS epidemic has contributed to an abrupt increase of the frequency of Kaposi sarcoma (KS), especially in Southern Africa. There is a need to describe the clinical features of this disease, its management, and its outcome in HIV positive children in Southern Africa. The aim of the study is to describe two different populations with HIV and KS from two African hospitals in Namibia and South Africa. *Material and Methods*. A retrospective descriptive study of patients with KS who presented to Tygerberg Hospital (TH) and Windhoek Central Hospital (WCH) from 1998 to 2010. Demographic data, HIV profile, clinical picture of KS, and survival were documented. *Results*. The frequency of KS declined from 2006 to 2010 in TH but showed an increase in the same period in WCH. Children in TH were diagnosed at a much younger age than those in WCH (44.2 months versus 90 months). Cutaneous lesions were the most common clinical presenting feature, followed by lymphadenopathy, intrathoracic and oral lesions. *Conclusions*. The clinical characteristics of KS in South Africa and Namibia differ in many aspects between the 2 countries.

## 1. Introduction

Kaposi Sarcoma (KS) is a low-grade malignant vascular tumor, with a viral etiology. The typical skin lesions were first described in 1872 [1] by Moritz Kaposi, a dermatologist from Hungary. While in most of the world the tumour was seen only sporadically, in regions from Central and Southern Africa it was found to be endemic, with incidence comparable to those of the cancer of the colon in resource-rich countries [2]. A dramatic increase in the incidence of this malignancy worldwide was associated with the HIV-AIDS epidemic, to the extent where KS became one of the AIDS-defining diseases. To distinguish it from the other known forms of the disease, that is, sporadic and endemic, when associated with AIDS, KS was said to be epidemic. The etiological agent, KS herpes virus (KSHV), was only discovered in 1994, as a result of the work by Chang and collaborators [3], who isolated and characterized the agent from tissue samples of epidemic KS.

KSHV is endemic in Central and Southern Africa. In South Africa, a study of seroprevalence of the virus found that 15.9% of healthy children were infected, while in those living with HIV the prevalence was 28.6% [4]. This prevalence explains why, with the advent of the HIV epidemic, the incidence of KS soared to previously unknown levels. The odds ratio for the association of HIV-Kaposi sarcoma in Malawian children was 93.5 (95% CI 26.9 to 324.4, *P* < 0.001) [5].

The literature on epidemic KS in African children is scarce; the disease in children is not as well described as in adults, and the diagnosis, treatment, and prognosis are insufficiently studied. This situation can be attributed to the rarity of specialized pediatric oncology services in Southern Africa, where the AIDS epidemic generates most of the cases of KS.

In South African children, an increase was seen from 1998 where one case was diagnosed to 2008 where 26 cases were documented in the South African Children's Cancer Registry (see Table 1).

KS is recognized as an AIDS-defining disease due to the strong association between the two entities. Since more than 3 million children with HIV were living in Sub-Saharan Africa in 2010, [6], the burden of KS is high in this region. The incidence of HIV in Namibia and South Africa is comparable [7], but both Namibia and South Africa are struggling to meet the need for ARV therapy in children [8, 9]. South Africa will fall short of achieving the Millennium Development Goals with child mortality rates increasing after the layout of these goals [10].

By the middle of 2011, the number of patients receiving antiretroviral (ARV) therapy in SA had increased to 1.79 million. In 2004 there were 4200 children (below 15 years) on ARV therapy, compared to 152 000 in 2011 [11]. The relative low rates of ARV therapy initiation in children are probably attributed to lower rates of HIV testing in paediatric population [12]. There is a disproportionately high incidence of HIV infection in the Sub-Saharan region. With the increase of ARV therapy availability, however, secondary malignancies are on the decline and the incidence of KS is expected to decrease as well [13].

The incidence of childhood cancer in South African children aged 0–14 years ranged between 33.4 and 47.2 per million from 2003 to 2007 [14], while in Namibia the incidence was slightly lower at 29.4 per million [15]. Many more children are diagnosed with cancer in developed countries, leading to a much higher incidence, of 176 per million, in the United States in 2005 [16]. Since the risk of developing a malignancy increases with HIV infection, it was expected that the incidence of childhood cancer would soar in countries with a high HIV burden, but this is not reflected in the available epidemiological data from South Africa [17]. There is very little research published on childhood cancers in Namibia and also a paucity of studies about treatment and survival of KS in the paediatric population of Sub-Saharan Africa [13].

This study proposed to analyse and compare Kaposi sarcoma in children in two African hospitals: Tygerberg Hospital (TH) in South Africa and Windhoek Central Hospital (WCH) in Namibia. Differences between the 2 groups of children diagnosed with KS were looked into in order to provide better insight into the characteristics of the disease. The variables of patient demographics, frequency of KS, comorbidities, management, survival, and HIV profile were assessed to find differences in this complex disease in the Southern African region.

## 2. Patients and Methods

### 2.1. Site and Study Period

The study evaluated all cases of KS in two hospitals in the Southern African region: Tygerberg Hospital (TH) situated in Cape Town in the Western Cape province of South Africa and Windhoek Central Hospital (WCH) in the neighboring Namibia, from January 1, 1998 until December 31, 2010.

### 2.2. Setting

Tygerberg Hospital (TH) is situated in the Western Cape province of South Africa, serving a population of 3.6 million people. It is the largest hospital in the Western Cape province of South Africa and the second largest hospital in the country [18]. The Department of Paediatrics has 308 beds and a dedicated hematology oncology unit where an average of 50 new patients are admitted yearly. All children diagnosed with a malignancy in the hospital drainage area are referred from peripheral hospitals and clinics to this tertiary center.

A dedicated paediatric tumor registry has been in existence for 25 years in South Africa, collecting data from all cases of malignancy nationwide. Data is sent from all pediatric oncology units and the private sector in the country to the tumor registry [14]. All units have ethical approval to collect and submit data to the registry through a combined national ethics agreement.

The prevalence of HIV in children and adults in the Western Cape province was 3.8% in 2008. The prevalence of HIV in children (0–15 years) in South Africa was estimated to be 2,5% in the same year [19].

WCH is the only government hospital in Namibia where childhood cancer cases are treated. There is no dedicated pediatric oncology tumor registry in Namibia, but the unit collects data of all new pediatric malignancies presenting to them.

In 2008, the paediatric oncology units from both hospitals decided to enter into a twinning agreement [20] leading to close cooperation, which includes discussion of new patients, adaption of protocols for specific types of malignancies, and uniformity of treatment regimens, addressing drug availability and sharing of data. Both units use similar protocols of chemotherapy for the treatment of KS.

### 2.3. Study Population

The Tygerberg Children's Cancer registry was used to obtain information regarding newly diagnosed KS in children treated in TH. Data of children treated for KS in WCH were obtained from the Namibian tumour registry.

### 2.4. Inclusion Criteria

All children aged 0–15 years with a diagnosis of KS were included in the study.

### 2.5. Exclusion Criteria

Patients older than 15 years were not included.

Patient folders were reviewed and the following information was extracted: age, sex, ethnic group, HIV status, date of diagnosis of HIV disease, degree of immunosuppression (WHO criteria), use of antiretroviral therapy, CD_4_ count, HIV viral load values, and date last seen or date when the patient died. HIV infection was confirmed via the enzyme-linked immunosorbent assay for HIV-specific antibodies or PCR-HIV testing in those children below the age of 18 months. The clinical picture of KS including symptoms, signs, site of disease, histological results, and radiological investigations was recorded, as well as any comorbid conditions, either at time of diagnosis or developing thereafter. Uniformity of histological evaluation was assured, since samples were processed by pathologists of the National Health Laboratory Service (NHLS) in South Africa and Namibia where similar laboratory protocols are applied. The modality of treatment used (chemotherapy, ARV therapy, radiotherapy and/or surgery) was documented.

### 2.6. Data Management

The data from patients fulfilling inclusion criteria were collected on a specifically designed data collection form and entered into a Microsoft Access database. Confidentiality was assured since the data provided to the principal investigator was anonymous.

### 2.7. Statistical Analysis

Treatment outcomes were analyzed using Statistica software using descriptive analysis and survival analysis by way of Kaplan-Meier curves. A *P* value of 0.05 or less was regarded as significant.

### 2.8. Ethical Considerations

Approval was obtained from the relevant Human Ethics Research Committees of the 2 participating centers (Institutional Review Board Number: IRB0005239). As this was a retrospective study, consent to access patient files was obtained through the custodian of the data and not through individual patient consent.

## 3. Results

The records of 21 patients were reviewed: 13 from TH and 8 from WCH.

### 3.1. Number of New Cases Diagnosed

**Figure 1:**
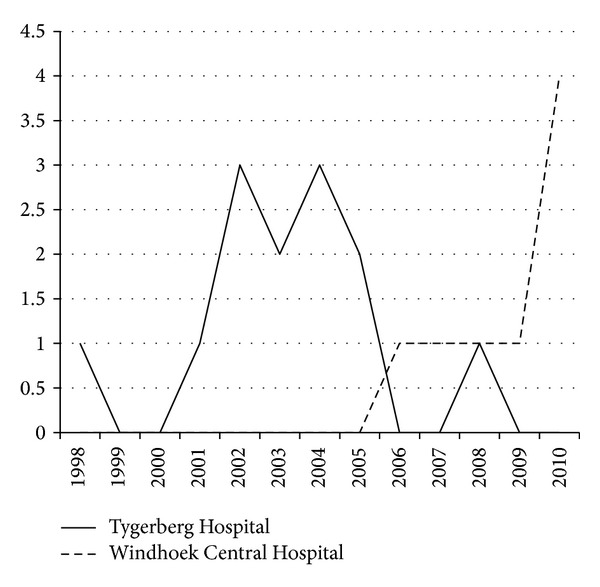
Number of cases newly diagnosed with Kaposi sarcoma in TH and WCH from 1998 to 2010.

In the TH cohort, one case was diagnosed in 1998, with a period of no new cases for 2 years. Most cases were diagnosed during 2000 to 2006, with one more child being diagnosed in 2008. From 2009 onwards, no new cases were seen until the end of the study period. In the WCH group, the first case was seen only in 2005. Thereafter one case per year was diagnosed, until 2010 when 4 new cases were identified (Figure 1).

The mean age at diagnosis in TH was 44.2 months (range 10 to 89 months) and in WCH 90 months (range 43 to 141 months).

### 3.2. Clinical Features

The commonest sign of disease was skin discoloration (38%; 10/26) with 5 cases each from TH (38%) and WCH (62.5%). Lymphadenopathy as the presenting feature was documented in 6 (23%) cases. The third most common presenting symptom or sign was respiratory distress due to intrathoracic KS lesions (4 patients; 15.3%). Two patients presented with oral lesions (7.6%), 1 with stridor (3.8%), and 1 with a neck abscess (see Table 2). Some patients presented with a combination of symptoms and signs.

The skin was the commonest site of disease with more than half of the patients presenting with skin lesions (13 patients). No specific skin area was more often seen than others. Lymph node involvement [6] was the second most common, followed by intraoral lesions/salivary gland involvement [5], intrathoracic lesions [4], and 1 case where the cranium was involved (see Table 3).

The diagnosis of KS was confirmed histologically in 76.9% of cases in TH and 88% in WCH by the NHLS laboratory services. In the remaining cases the diagnosis was made clinically.

No data was available regarding KSHV status as testing was not routine practice at these hospitals during the study period.

All children in the study were HIV positive. In the TH cohort, 62% (8 patients) were already on ARV therapy at the time of the diagnosis, while 38% of the WCH group were receiving ARV therapy at diagnosis. Although KS is a WHO stage IV defining disease, 27% of children in TH and 87.5% in WCH were already classified as having stage IV disease prior to the diagnosis of KS.

Radiological changes ascribed to KS were documented in 2 cases in the Tygerberg group: one with bilateral pleural effusions and one with pulmonary and pleural infiltrations. Both cases were histologically confirmed. In WCH, radiological changes were documented in only 1 case of disseminated KS (right lower lobe opacification and pleural effusion).

### 3.3. Comorbid Disease

Pulmonary tuberculosis (PTB) was the predominant comorbid disease present in 38.4% (*n* = 5) of the TH group. No cases of PTB were found in the WCH population. PTB was diagnosed by a combination of symptoms, chest radiographic changes, and sputum or gastric aspirate microscopy and culture.

### 3.4. Treatment and Outcome

Both TH and WCH used similar chemotherapy protocols comprising vincristine (1.5 mg/m^2^), adriamycin (20 mg/m^2^), and bleomycin (15 iU/m^2^) given at 3-4-week intervals for a total of 6 cycles. Local therapy is instituted in cases of limited mucocutaneous disease, but no case in this study required control locally. Paclitaxel was not used in any child in this study as no case of relapsed KS was diagnosed.

### 3.5. Survival

The survival in WH was 62.5% and in TH 46,1%. In the combined study population 10 patients (48%) died after an average of 2.4 months. The children that survived (*n* = 11) were followed up for a mean period of 14 months. In the Tygerberg group 3 deaths were attributed to respiratory insufficiency (intrapulmonary bleeds, direct intrathoracic tumor infiltration, and chylothorax). In the WH group the causes of death were not identified.

In the TH group 2 children suffered disease relapse, 6 months and 29 months after the initial diagnosis. In the first case of early relapse it was decided to continue with symptomatic treatment only and not to give chemotherapy. This child never received ARV therapy and died due to complications of infection. The second patient relapsed in a different lymph node site. Chemotherapy was recommenced (rescue protocol) and the patient was alive with disease during the last followup in 2012.

## 4. Discussion

There are many differences in the clinical presentation of KS in Southern Africa, notably in the age of the children at diagnosis, frequency between the 2 study populations, and profile of HIV between the 2 countries. Similarities however exist in, for example, clinical presentation and gender distribution, which are also in keeping with the KS that is described in children in the rest of Africa [21, 22].

Twenty-one cases of KS were identified in these 2 African centers over the time period from 1998 until 2010. Children were diagnosed at a later age in the Windhoek group. This could reflect a missed group of children with KS at a younger age in the Namibian population, who did not benefit from screening programs to facilitate early diagnosis. The majority of patients in the Tygerberg group were diagnosed before 2006, in contrast to an increase in KS cases in WCH from 2006 onwards. This difference in patterns could be explained due to a difference in access to ARV therapy between the 2 study groups. Another possibility is that increased cancer awareness and screening programs could have led to more cases diagnosed in Namibia since 2005 as well as better referral of HIV positive children to monitor HIV associated comorbidities and access to ARV therapy.

The annual incidence of cancer in South African children aged 0–14 years is estimated to be between 33.4 and 47.2 per million from 2003 to 2007 [14]. In a recent unpublished study in the same age group, it was found that the incidence in Namibian children is 29.4 per million [15], although this might be an underestimation due to the lack of a formal or consistent cancer registry in Namibia. These figures are much lower than those in developed countries where the incidence of cancer in children in the United States in 2005 was estimated to be 176 per million [16]. It is likely that a large proportion of childhood cancers are not diagnosed in the Sub-Saharan African region.

In TH between 1998 and 2010 there were 547 newly diagnosed patients with a malignancy (average of 42 malignancies/year). KS represented 2.8% of all malignancies in TH during this period. This is less than the national South African data where KS comprised almost 5% of the total pediatric malignancies in 2008 [17]. National data showed a progressive increase in KS frequency in 1998 from 1 patient into the next decade to 28 patients. In Namibia from 2003 to 2010, 191 new pediatric malignancies were diagnosed, of which 11 cases (5.7%) were KS [15], similar to the national South African data.

This apparent discrepancy in frequency of KS has to be correlated with the incidence and management of HIV in both populations. In South Africa the HIV prevalence in children below 14 years decreased from 5.6% in 2002 to 2.5% in 2008 [17]. This considerable reduction is largely attributed to the national Prevention of Mother to Child Transmission (PMTCT) program and the involvement of the South African government health services. The Western Cape is the province in South Africa with the lowest prevalence of HIV infection (in patients ≥ 2 years) at 1.9% in 2005 [18]. In Namibia the prevalence of HIV infection in children below 14 years was estimated to be 2.1% in 2010/2011 [7]. Estimates of the HIV epidemic in Namibia in 2008 have suggested that Namibia has not yet reached its peak prevalence, but it did have a rapid prevalence growth period, which ended in 2001. In 2007/2008 77% of new infections were among women aged 15–24 years [23], which is a critical area for the country to address in Prevention of Mother to Child Transmission.

The South African national antiretroviral treatment program started in 2004 and has steadily grown. Inequities do exist between urban and rural areas in the country and there are also interprovincial differences in achieving ARV therapy for all in need. In 2010 UNICEF estimated that 54% of children who were living with HIV in South Africa were receiving ARV therapy [24]. In the Western Cape in 2005, access to ARV therapy varied from 68 to 85% between primary care clinics and tertiary hospitals [25].

Coverage of ARV therapy in Namibia increased rapidly from 3% (2003) to almost 60% (2007) according to national published data [26], but the majority of children in this study were already classified as WHO stage IV at the time of diagnosis. This might represent a missed group of children, which were not accessing ARV therapy from an early age.

In a review of early infant testing of HIV in Namibia and 3 other countries, Chatterjee et al. [27] reported a very high rate of loss to followup in Namibia in the postnatal population who tested HIV-PCR positive. This could in part be an explanation of the difference in age at presentation of KS between WCH and TH. Our study found that children from WCH were being diagnosed at almost double the mean age of children in TH.

The mainstay of systemic treatment for KS is chemotherapy and ARV therapy. Local treatment modalities comprise intralesional vincristine, intralesional interferon alpha, radiotherapy, and surgery in selected cases [28]. In Sub-Saharan Africa access to treatment poses more challenges and there are relatively few studies on treatment response in this region [13].

Both groups had a significant male predominance, corresponding to results obtained by Amir et al. in Tanzania [29]. As in the other African studies, this predominance also tended to be stronger in younger children [30]. This male predominance remains unexplained in our study and indeed in Africa.

Site of presentation of lesions was similar to that found in other African studies. A Ugandan study of 73 children found increased lymph node presentation despite higher CD_4_ values. In the South African literature there is a strong focus on site of presentation. A small case series of 6 South African children described the radiological findings of pulmonary KS as an initial site of presentation [31]. Other series looked at frequency of skin lesions in children with KS and found that younger children frequently have lymphadenopathy as presenting feature [22]. This study confirmed the importance of nodal lesions as presenting feature, but cutaneous lesions were still predominant in both study groups.

This study looked at the difference in clinical presentation in 2 populations also differing in HIV and ARV therapy profile. Of note is a possible inverse relationship between the use of ARV therapy and the frequency of KS. As to the clinical expression of KS there are similarities that also correlate well with those seen in the rest of Africa.

## 5. Conclusion

KS is a major AIDS-defining disease in Sub-Saharan children. KS in TH and WCH differs in many aspects notably age at diagnosis, where children were diagnosed at double the age in the Windhoek group. The number of new cases diagnosed was found to increase over the study period in WCH while a decrease was seen in the TH group. This could reflect the difference in access to ARV therapy in the study populations. A high mortality rate was found in the combined study group, probably reflecting the initial lack of availability of ARV therapy. The widespread use of ARV therapy currently is making major changes in the mortality rate. An opportunity exists for prospective studies to find the best modality of treatment for KS in the Sub-Saharan region.

## 6. Limitations

Completeness of the study was limited by incomplete record keeping particularly chemotherapy use in the Namibian population group which used the same treatment protocol as TH, but it was not always strictly followed. As this is a retrospective analytical study, some missing information can be expected. Followup was incomplete in many patients due to patient adherence factors, as expected in a third world setting. The small number of cases recorded overall might not be representative of the entire population. In Namibia it is estimated that 75% of childhood cancer cases are not seen at the paediatric oncology unit. A few cases are seen privately and not documented, but in all likelihood the majority is missed, which would have a big influence on the frequency.

## Figures and Tables

**Table 1:** Annual numbers of KS recorded in South African Children's Cancer Registry 1998–2008, mean age, and survival to date.

Year	Total cancers	KS	% of total cancers	Mean age (months)	Alive	% survival
1998	592	1	0.16	62	0	0
1999	575	1	0.17	56	1	100
2000	716	9	1.2	54.3	1	11
2001	648	12	1.8	66.6	10	83
2002	599	14	2.3	78.2	5	35
2003	643	20	3.1	66	13	65
2004	723	24	3.3	79.3	16	66
2005	650	24	3.6	104.1	10	41
2006	592	16	2.7	82.5	13	81
2007	564	13	2.3	82.7	12	92
2008	529	26	4.9	71.7	18	69

Total	6302	160	2.5	73.3	99	61

**Table 2:** Distribution of signs and symptoms in the cases studied.

Symptom or sign	Namibia	Western Cape	Total
Skin discoloration	5	5	10 (38.4%)
Lymphadenopathy	2	4	6 (23%)
Respiratory distress/shortness of breath	1	3	4 (15.3%)
Oral lesions	1	1	2 (7.6%)
Stridor	0	1	1 (3.8%)
Abscess	1	0	1 (3.8%)
Other	1	1	2 (7.6%)

**Table 3:** Anatomical distribution of lesions (some patients presented with more than 1 site involvement).

Site of lesions	Namibia	Western Cape	Number of cases
Skin			
Facial	2	1	3 (10.3%)
Trunk	0	2	2 (6.8%)
Abdomen	0	2	2 (6.8%)
Leg/foot	2	1	3 (10.3%)
Generalised skin/other	1	2	3 (10.3%)
**Total skin**	**5**	**8**	**13 (44.8%)**
Intrathoracic	1	3	4 (13.8%)
Lymph nodes	2	4	6 (20.7%)
Intraoral/salivary gland	2	3	5 (17.2%)
Cranium	0	1	1 (3.4%)

	Total: 29		
